# Clinicopathological Features and Prognosis of Indonesian Patients with Gliomas with *IDH* Mutation: Insights into Its Significance in a Southeast Asian Population

**DOI:** 10.31557/APJCP.2020.21.8.2287

**Published:** 2020-08

**Authors:** Rusdy Ghazali Malueka, Ery Kus Dwianingsih, Halwan Fuad Bayuangga, Andre Stefanus Panggabean, Ibnu Widya Argo, Aditya Dwi Donurizki, Sabillal Shaleh, Adiguno Suryo Wicaksono, Kusumo Dananjoyo, Ahmad Asmedi, Rachmat Andi Hartanto

**Affiliations:** 1 *Department of Neurology, Faculty of Medicine, Public Health and Nursing, Universitas Gadjah Mada, Dr. Sardjito General Hospital, Yogyakarta, Indonesia. *; 2 *Department of Anatomical Pathology, Faculty of Medicine, Public Health and Nursing, Universitas Gadjah Mada, Dr. Sardjito General Hospital, Yogyakarta, Indonesia. *; 3 *Division of Neurosurgery, Department of Surgery, Faculty of Medicine, Public Health and Nursing, Universitas Gadjah Mada, Dr. Sardjito General Hospital, Yogyakarta, Indonesia. *

**Keywords:** IDH, glioma, clinicopathological features, prognosis, Indonesia

## Abstract

**Background:**

Gliomas remain one of the most common primary brain tumors. Mutations in the isocitrate dehydrogenase (*IDH*) gene are associated with a distinct set of clinicopathological profiles. However, the distribution and significance of these mutations have never been studied in the Indonesian population. This study aimed to elucidate the association between *IDH* mutations and clinicopathological as well as prognostic profiles of Indonesian patients with gliomas.

**Methods:**

In total, 106 patients with gliomas were recruited from a tertiary academic medical center in Yogyakarta, Indonesia. Formalin-fixed paraffin-embedded and fresh tissue specimens were obtained and sectioned for hematoxylin-eosin staining and immunohistochemical examinations. Genomic DNA was isolated and analyzed for the presence of *IDH* mutations using standard polymerase chain reaction and nucleotide sequencing methods. Clinicopathological data were collected from medical records.

**Results:**

Although no *IDH2* mutation was identified, *IDH1 *mutations were found in 23 (21.7%) of the patients. Patients with *IDH1* mutations tended to have a history of smoking and a shorter interval between onset of symptoms and initial surgical interventions. Frontal lobe involvement, oligodendroglial histology, lower Ki67 expression, WHO grades II and III gliomas, and methylated O6-methylguanine-DNA methyltransferase (MGMT) promoters were significantly associated with the presence of *IDH1* mutations. Compared with patients with *IDH1*-wild-type, patients with *IDH1* mutation were observed to have a longer overall survival.

**Conclusions:**

*IDH1* mutations are associated with certain clinicopathological and prognostic profiles in Indonesian patients with gliomas. This finding demonstrates the importance of identifying *IDH* mutations as part of the management of patients with glioma in Indonesia.

## Introduction

Gliomas are the most common primary tumors in the central nervous system (CNS) in adults. They encompass a group of tumors that share histological features with normal glial cells, i.e., astrocytes, oligodendrocytes, and ependymal cells. The annual incidence in Caucasians and Asians has been estimated to be 6 cases per 100,000 people (Hofer et al., 2014). Among those, approximately 80% were diagnosed with malignant gliomas (Alifieris and Trafalis, 2015).

Formerly, the World Health Organization (WHO) classification of CNS tumors was based on histological and degrees of malignancy (Alentorn et al., 2015; Alifieris and Trafalis, 2015). The 2016 edition, however, has incorporated molecular parameters into the classification criteria. By integrating both genotypic and phenotypic profiling, it was hoped that it would add a level of objectivity and yield more biologically homogenous diagnostic entities. This, in turn, should lead to improved diagnostic accuracy and patient management (Louis et al., 2016). 

Despite the administration of intensive therapies, the outcomes of patients with glioma, especially those with glioblastoma multiforme (GBM), remain poor. A median progression-free survival (PFS) of as short as 6.9 months and median overall survival (OS) of 14.6 months have been reported in patients with GBM even after they underwent surgery plus standard concomitant chemoradiotherapy (CCRT) (Qi et al., 2011). Therefore, a novel therapeutic strategy based on a deeper understanding of tumors’ biological characteristics is urgently needed. 

One of the molecular biomarkers of significant interest for gliomas is isocitrate dehydrogenase (*IDH*) mutation (Yang et al., 2015). Most of the reported *IDH* mutations were missense heterozygous mutations at codon 132 of the *IDH1* gene and codon 172 of the *IDH2* gene (Turkalp et al., 2014). Approximately 90% of patients with *IDH1/2* mutations carry the single nucleotide change c.395G>A in the *IDH1* gene. Approximately 3% of all *IDH1/2* mutations are mutations in *IDH2 c.515G> A (R172K)* (Horbinski et al., 2009). *IDH1* R132 mutations have been found to occur in 55-80% of grade II and III oligodendrogliomas and astrocytomas.* IDH1* mutations are rare in primary GBM (<10%) but are frequently observed in secondary GBM (>80%). These mutations are associated with a good prognosis (van den Bent et al., 2010; Uno et al., 2011). Mutations of the *IDH2* gene are less common, with the most frequent type of tumor being oligodendroglial (Raynaud et al., 2010).

*IDH* gene encodes three types of IDH enzymes, namely IDH 1, 2, and 3. These enzymes convert isocitrate to alpha-ketoglutarate (α-KG) and NADPH, which normally function to protect cells against oxidative stress. *IDH* mutations cause failure of gene functions, leading to a decrease in α-KG production with a subsequent increase in D-2-hydroxyglutarate *(D-2HG*) production. *D-2HG *has a structure similar to α-KG and competitively inhibits the activity of various dioxygenase enzymes. This then contributes to the pathogenesis of gliomas. *D-2HG* is also known to induce vascular endothelial growth factor (VEGF) expression that promotes angiogenesis and tumor growth (Kloosterhof et al., 2011; Ichimura, 2012).

Studies have shown that tumors with *IDH1/2 *mutations have better outcomes, with a median OS of 31 months in GBM patients with these mutations as compared to 15 months in GBM patients without the mutations. In the case of anaplastic astrocytoma, the median OS in patients with *IDH* mutations was 65 months, while without the mutation, it was reported to be as short as 20 months (Lee et al., 2007; Fu et al., 2010; Kloosterhof et al., 2011). GBM patients with *IDH1* mutations have a more favorable response to temozolomide chemotherapy than those without mutations. Thus, the presence of *IDH1/2 *mutations can be a useful predictor for treatment success, and the testing for these mutations has become part of standard clinical practice (Krell et al., 2013).

It is clear that the detection of* IDH1/2* mutations is necessary to understand glioma pathogenesis and tailor a suitable therapy for patients. Therefore, this study aimed to elucidate the association between *IDH* mutations and clinicopathological as well as prognostic profiles of Indonesian patients with gliomas.

## Materials and Methods

*Patients and samples*

The study included both retrospective and prospective samples. A total of 56 glioma samples from patients treated from 2010 to 2017 were enrolled. These samples were obtained from the tissue bank at a tertiary academic medical center (Dr. Sardjito General Hospital) in Yogyakarta, Indonesia. Additionally, 50 newly diagnosed glioma patients from October 2017 to April 2019 were recruited as prospective samples. Hematoxylin-eosin stained tissue slides were assessed by an experienced neuropathologist blinded to the *IDH1/2* mutation status and classified according to the 2016 WHO classification of CNS tumors. Detailed demographic and clinical data were collected from medical records accordingly. This study was approved by the Institutional Review Board (IRB), Universitas Gadjah Mada, Indonesia. Written informed consents were obtained from patients themselves or their family members. All methods were done according to the relevant guidelines and regulations of Universitas Gadjah Mada IRB and Indonesian Ministry of Health. 

*DNA extraction*

Genomic DNA was extracted from formalin-fixed paraffin-embedded (FFPE) and fresh tissue specimens for retrospective and prospective samples, respectively. For FFPE tissue specimens, following deparaffinization of unstained tissue sections, DNA was isolated using the QIAamp DNA FFPE Tissue Kit (QIAGEN, Cat. #56404, Hilden, Germany) according to the manufacturer’s instructions. DNA from fresh glioma tissue was extracted using the Quick DNA FFPE MiniPrep Kit (Zymo Research, USA). The NanoVue Plus spectrophotometer (GE Healthcare Life Sciences, Pittsburgh, PA, USA) was then used to assess the quantity of recovered DNA.

*PCR amplification and sequencing analysis*

Polymerase chain reaction (PCR) was performed to amplify the codons R132 and R172 of the exon 4 of the *IDH1* and *IDH2* genes, respectively. The* IDH1* forward (5’-ACC AAA TGG CAC CAT ACG A-3’) and reverse (5’-GCA AAA TCA CAT TAT TGC CAA C-3’) primers generated a 130 base pair (bp) PCR product while the *IDH2* forward (5’-GCT GCA GTG GGA CCA CTA TT-3’) and reverse (5’-TGT GGC CTT GTA CTG CAG AG-3’) primers generated a 293 bp PCR product (Arita et al., 2014). 

PCR reactions were set at a total volume of 25 µL containing 2 µL of genomic DNA, 3.45 µL of Taq DNA Polymerase (Invitrogen, Thermo Fisher Scientific, Cat. #10342020, Waltham, MA, USA), 0.2 µL of dNTP (Thermo Scientific, Thermo Fisher Scientific, Cat. #R0191, Waltham, MA, USA), and 1 µL of each primer. The PCR was performed using Veriti Thermal Cycler (Applied Biosystem, Thermo Fisher Scientific, Cat. #4375786, Waltham, MA, USA) under the following conditions: initial denaturation at 95^o^C for 2 minutes followed by 40 cycles of denaturation at 95^o^C for 30 seconds, annealing at 53^o^C (*IDH1*) or 56^o^C (*IDH2*) for 30 seconds, and extension at 72^o^C for 2.5 minutes. The amplified products were then visualized using 2% agarose gel electrophoresis to confirm the presence of a single band of the predicted size. After purification with MinElute PCR Purification Kit (QIAGEN, Cat. #28004, Hilden, Germany), sequencing of the products in both sense and antisense directions was performed using the BigDye Terminator v3.1 Cycle Sequencing Kit (Applied Biosystem, Thermo Fisher Scientific, Cat. #4337455, Waltham, MA, USA). Each sample was categorically defined as positive or negative for *IDH1/2* gene mutation based on the sequencing results.

*Methylation-specific qRT-PCR*

Bisulfite conversions of genomic DNA to convert unmethylated cytosine to uracil were performed using the EZ DNA Methylation-Gold Kit (Zymo Research Cat o. D5005 Lot No. ZRC 200814). Up to 2,000 ng of DNA was treated to obtain adequate converted DNA for MGMT promoter methylation status analysis using a quantitative real-time PCR method as described elsewhere (Rivera et al., 2010). 

*Immunohistochemistry*

Expression of* Ki67* was detected by immunohistochemistry staining using antibody for Ki-67 (SP6, rabbit monoclonal antibody, BioCare Medical, Cat. #OAI325T60, Pacheco, CA, USA). A trained pathologist blinded to the* IDH1/2* mutation status examined the percentage of positively-stained tumor cells (Zhang et al., 2013). 

*Surveillance and follow-up*

Survival data were collected in the outpatient clinic during patients’ visits, in the ward during patient hospitalization periods, and through phone call or home visit. OS was calculated as the time between the first surgical intervention and death or last follow-up (for censored cases). 

*Statistical analysis*

The associations between *IDH* gene mutation and clinicopathological features were analyzed. Numerical data are presented as mean and were interpreted using T-test or Mann-Whitney test accordingly. Meanwhile, categorical variables are presented as proportions and were analyzed using Chi-square or Fisher’s exact test. Survival distributions for each group were plotted using the Kaplan-Meier method and compared statistically using the log-rank tests. The reported p values are 2-sided, and p < 0.05 was considered to be statistically significant.

## Results

**Figure 1:**
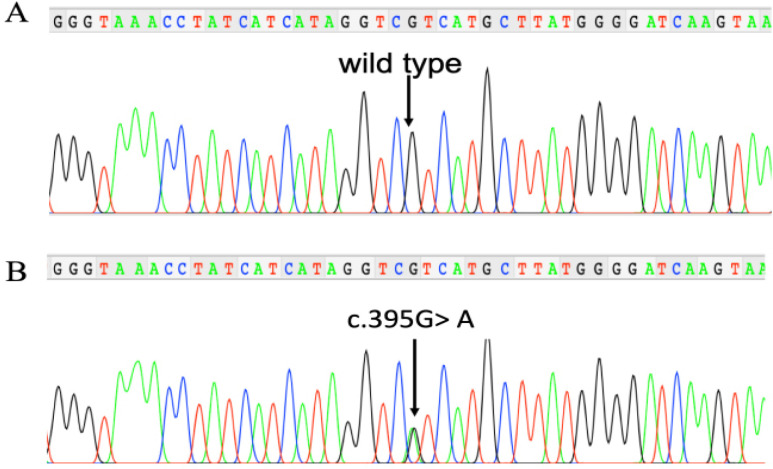
Nucleotide Sequencing Results Showing Wild-Type (A) and Mutant (B) *IDH1* Genes

In total, 106 patients were included in this study. The demographic and clinical characteristics of the patients are shown in Table 1. Among 106 patients, 23 (21.7%) of them carried *IDH1* mutation as shown by Sanger sequencing (Figure 1). Among this group, 22 were positive for *R132H *mutation, while one patient was found to harbor an *R132G* mutation. For further analysis, both mutations were categorized into the IDH1-mutant group. No *IDH2* mutations were found. The mean age of the patients was 37.6±18.6 years old. There were 58 (54.7%) men and 48 (45.3%) women included in this study. There were no differences in mean age and sex distribution between *IDH1-*mutant and *IDH1*-wild-type groups (p>0.05). A higher proportion of patients with a history of smoking, however, was found in the *IDH1*-mutant compared to the *IDH1*-wild-type group (57.1 % vs. 23.8% respectively, p=0.022).

The mean Karnofsky Performance Status (KPS) scale before surgery was 53.2±18.1. The IDH1-mutant and wild-type groups had similar KPS on admission (54.7±17.7 vs. 52.9±18.3, respectively, p=0.755). About 30% of the patients reported a symptom of seizures. There was no association between the occurrence of seizures and *IDH1* mutation (p=0.226). 

A significant association was found between* IDH1 *mutation and tumor location. Patients with *IDH1 *mutations had a higher proportion of frontal lobe involvement as compared to wild-type patients (56.25% vs. 27.4 % respectively, p=0.029). 

*IDH1 *mutations were also shown to be significantly associated with histological subtypes of glioma (p<0.001). Our study showed that 16.5% of astrocytomas harbored *IDH1 *mutation. This frequency was much lower compared to oligoastrocytomas (60%) and oligodendrogliomas (75%). No patient with ependymoma was positive for this mutation.

According to the degree of malignancy, the most commonly found glioma in decreasing order of frequency was grade IV (40.6%), followed by grade II (34%), grade III (16%), and grade I (9.4%). Most of the *IDH1 *mutations were found in grade II gliomas (56.5%). Percentages of samples with* IDH1* mutation were 36.1% in grade II glioma, 17.7% in grade III glioma, 14% in grade IV glioma, and 10% in grade I glioma. As much as 30.8% of grades II and III glioma harbor *IDH1* mutation compared to 13% in the grades I and IV glioma group. In total, grades II and III gliomas accounted for 69.6% of* IDH1* mutations. This was significantly higher than the proportion of mutation in grades I and IV (p=0.026). Expression of Ki67 was found to be significantly higher in the* IDH1-*wild-type group compared to the *IDH1*-mutant group (p=0.027). 

**Figure 2:**
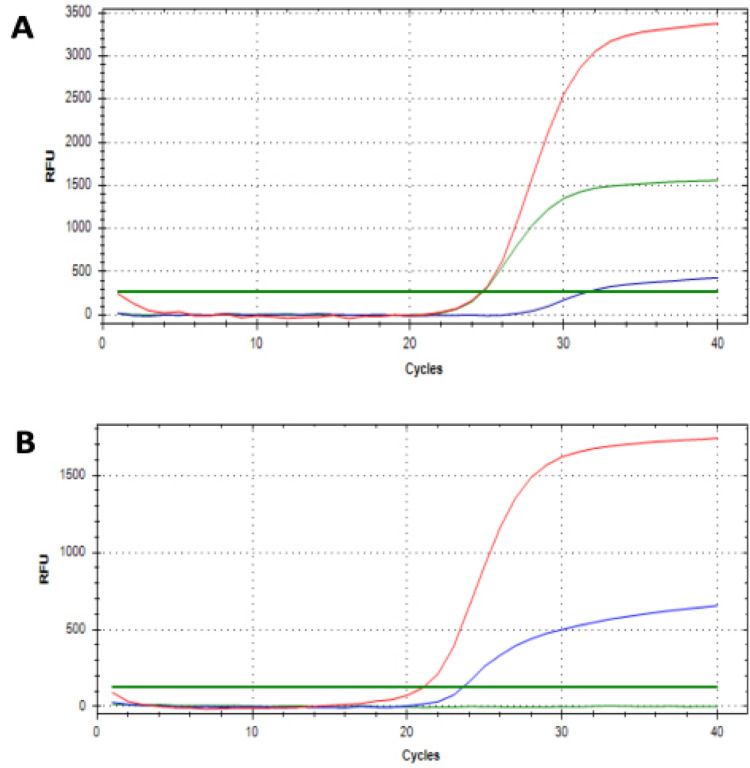
qRT-PCR Showing the Amplification of Methylated *MGMT* Promoter (Green curve), Unmethylated MGMT Promoter (Blue Curve), and a *COL2A1* Control (Red Curve). Examples of a glioma sample with (A) and without (B) *MGMT* promoter methylation

Due to the limited quantity of retrospective samples, *MGMT* methylation analysis was performed in prospective samples only. Two samples were excluded from further analyses as they did not allow for adequate interpretation of methylation-specific qPCR results. Among 48 samples analyzed, 12 (25%) of them showed methylation in *MGMT* promoters (Figure 2). IDH1-mutants showed a significantly higher proportion of *MGMT* methylation compared to the wild-type group (55.6% vs 18%, p=0.032). 

**Figure 3:**
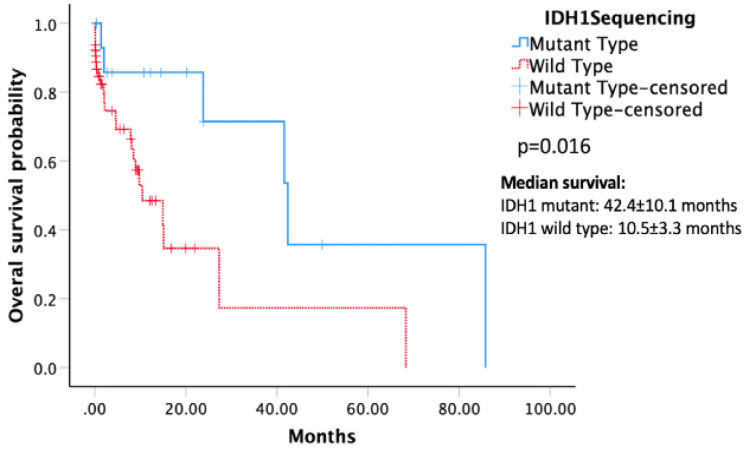
Kaplan-Meier Survival Analysis Demonstrating that *IDH1*-mutant Glioma Patients (Straight Line) had a Significantly Longer Overall Survival (p = 0.016; log-rank test).

Patients without *IDH* mutations had a significantly shorter interval between onset of symptoms and initial surgical interventions compared to the patients carrying the mutation (mean 4.64±6.8 months vs. 13.3±16.6 months, p=0.025). Kaplan-Meier survival analysis demonstrated that patients with *IDH1* mutations had a longer OS compared to those in the wild-type group (median OS 42.4±10.1 vs. 10.5±3.3 months) (Figure 3). A significant p-value of 0.016 for the difference in OS was obtained through log-rank tests. 

**Table 1:** Subject Characteristics (n=106)

Variable	Total	*IDH1*-wild-type	*IDH1*-mutant	*P*-value
Number of patients, n (%)	106 (100)	83 (78.3)	23 (21.7)	
Mean age, years (SD)	37.6 (18.6)	37.1 (20.1)	39.3 (12.1)	0.768^#^
Sex, n (%)				
Male	58 (54.7)	43 (51.8)	15 (65.2)	0.253
Female	48 (45.3)	40 (48.2)	8 (34.8)	
A history of smoking, n (%)				
Yes	23 (29.9)	15 (23.8)	8 (57.1)	0.022^##^
No	54 (70.1)	48 (76.2)	6 (42.9)	
Karnofsky Performance Scale pre-surgery (n=85), mean ± SD	53.2 (18.1)	52.9 (18.3)	54.7 (17.7)	0.755^#^
Reported symptom of seizure, n (%)				
Yes	25 (29.8)	18 (26.5)	7 (43.8)	0.226^##^
No	59 (70.2)	50 (73.5)	9 (56.2)	
Tumor location (include this lobe/not)				
Frontal	26/52	17/45	9/7	0.029
Temporal	36/42	27/35	9/7	0.364
Parietal	32/46	25/37	7/9	0.804
Occipital	7/71	6/56	1/15	1^##^
Others	16/62	15/47	1/15	0.169^##^
Histological type, n (%)				
astrocytic	85 (80.2)	71 (85.5)	14 (62.5)	<0.001^##^
mixed (oligoastroglial)	5 (4.7)	2 (2.4)	3 (12.5)	
oligodendroglial	8 (7.55)	2 (2.4)	6 (25)	
ependymal	8 (7.55)	8 (9.6)	0 (0)	
Grade, n (%)				
Grade I	10 (9.4)	9 (10.8)	1 (4.4)	0.091^##^
Grade II	36 (34)	23 (27.7)	13 (56.5)	
Grade III	17 (16)	14 (16.9)	3 (13)	
Grade IV	43 (40.6)	37 (44.6)	6 (26.1)	
Combined grade, n (%)				
Grade I & IV	54 (50.9)	47 (56.6)	7 (30.4)	0.026
Grade II & III	52 (49.1)	36 (43.4)	16 (69.6)	
Ki67, mean (SD), n=93	13.4 (13.3)	14.99 (13.97)	7.7 (8.99)	0.027^#^
MGMT promoter methylation, n (%)				
Methylated	12 (25)	7 (18)	5 (55.6)	0.032^##^
Unmethylated	36 (75)	32 (82)	4 (44.4)	
Interval between onset of symptoms to initial surgical interventions, months (SD) (n=84)	6.4 (10.1)	4.64 (6.8)	13.3 (16.6)	0.025^#^
Median overall survival, month (SD) (n=80)	23.8 (8.4)	10.5 (3.3)	42.4 (10.1)	0.016^###^

^#^, Mann-Whitney U test,^ ##^, Fisher’s exact test, ^###^, Kaplan-Meier with log-rank test, Chi-square tests were performed for the rest of the analyses

## Discussion

Our study showed that 21.7% of glioma patients harbored* IDH1* mutations. Previous studies in Asian populations revealed the frequency of *IDH1* mutations in Asian patients to range from as low as 7.8% in Japan to 74% in China (Yusoff et al., 2016). The studies were performed in the East and South Asian populations. A previous study in Southeast Asia was performed in Malaysia, showing the incidence of *IDH1* mutation at 35% (Yusoff et al., 2016). Their study, however, included all types of brain tumors, including meningioma and medulloblastoma. To our knowledge, our study is the first study to identify the incidence of *IDH1* mutation in glioma and the association with clinicopathological profiles in a Southeast Asian population. 

Our study did not find any significant associations between* IDH1* mutation and sex as well as age at the time of diagnosis. These findings were consistent with independent studies that also did not see any difference in relation to sex (Yan et al., 2012; Zhang et al., 2013; Yusoff et al., 2016). However, other studies showed that this mutation was associated with the age at diagnosis (Qi et al., 2014; Ohno et al., 2016). Patients with *IDH1* mutated glioma were reported to be younger than patients with wild-type* IDH1* gene (Yan et al., 2009, 2012; Molenaar et al., 2014; Qi et al., 2014; Ohno et al., 2016). The disagreement in this regard may be due to the mixture of different tumor types in the* IDH1-*wild-type group in our study. Presumably, the number of patients with *IDH1-*wild-type tumors with younger average age (i.e., pilocytic astrocytoma, ependymoma) was higher than that of those with primary GBM, thus lowering the total average age in this group.

Interestingly, our study found a significant association between *IDH1* mutation and smoking. Patients with *IDH1* mutation have a higher proportion of smokers compared to the group without the mutation (57.1% vs. 23.8% respectively, p=0.022). A previous study in patients with myelodysplastic syndromes (MDS) and chronic myelomonocytic leukemia (CMML) showed that smoking more than two packs per day or smoking for more than 40 years were associated with *IDH1 *mutations (Madanat et al., 2017). *IDH1/2 *mutations have also been associated with extensive smoking history in patients with lung adenocarcinoma (Toth et al., 2018). In the case of glioma, previous studies have failed to show an association between glioma risk and smoking status, intensity, duration, or age at smoking initiation (Holick et al., 2007). A meta-analysis of 24 cohort or case-control studies also revealed that cigarette smoking is not significantly associated with adult glioma in the overall population. They did find a small but statistically significant association in female smokers (Li et al., 2016). A more recent study from Korea, however, reported that current smokers had a higher risk to have malignant glioma compared to those who never smoked (Ahn et al., 2020). Several mechanisms have been proposed to explain this association. Smoking may induce multiple distinct mutational signatures, including* IDH* mutation, in tissues directly and indirectly exposed to smoking (Ahn et al., 2020; Madanat et al., 2017). Nicotine has also been shown to enhance the proliferation and migration of human glioma cells. Recently, the role of cigarette smoking in breaching the blood-brain barrier via the impairment of endothelial tight junctions has also been shown, possibly facilitating penetration of environmental carcinogens to the brain (Ahn et al., 2020). To the best of our knowledge, our study is the first study showing an association between *IDH1* mutation and smoking in patients with glioma. The fact that patients with *IDH1* mutation are more likely to be smokers can provide insight into the role of smoking in glioma formation, as *IDH1* mutation is a frequent, likely early event in gliomagenesis (Turkalp et al., 2014). The role of smoking in inducing *IDH* mutation, and the mechanisms behind it, require further study. 

This study did not show a significant difference in Karnofsky Performance Status (KPS) score on admission (before surgery). This agrees with previous studies, which also did not find a significant association between *IDH* mutation and KPS score (Qi et al., 2014). Previous studies have shown that *IDH1* mutation is independently associated with preoperative seizures (Chen et al., 2017; Li et al., 2018). The D-2-hydroxyglutarate (D2HG) product of mutant *IDH1 *has a similar chemical structure to glutamate, an excitatory neurotransmitter. D2HG is released by tumor cells into the microenvironment, activating neuronal NMDA receptors, therefore increasing neuronal activity. This leads to glioma-related epilepsy (Chen et al., 2017; Li et al., 2018). This association has been particularly shown in lower-grade glioma. However, the result for higher grade glioma has been inconsistent. Chen (2017) showed that the relationship between *IDH1* mutations and seizures did not depend on the grade of the glioma. Other studies have shown this association in GBM, but not in anaplastic glioma (Yang et al., 2014; Toledo et al., 2017; Li et al., 2018). Our study, which included glioma from all grades, did not show an association between seizures and *IDH1* mutation. 

This study showed that* IDH1* mutation is associated with the location of the tumor.* IDH1* mutated tumors were more frequently located in the frontal lobe (p=0.026). This is in accordance with previous reports, which also showed a higher proportion of frontal lobe tumor location in patients with *IDH1* mutation (Yan et al., 2012; Zhang et al., 2013; Qi et al., 2014). The underlying mechanisms of this association need further investigation. 

Our study showed that 16.5% of astrocytoma harbor *IDH1* mutation. This is much lower compared to the frequency of this mutation in oligoastrocytomas (60%) and in oligodendrogliomas (75%). No patients with ependymoma showed this mutation. Previous studies have indeed shown the association of *IDH1* mutation with histology and grading of gliomas (Yan et al., 2009; Qi et al., 2014; Yusoff et al., 2016; Wang et al., 2016; Deng et al., 2018). The percentage of oligodendroglioma samples with *IDH1* mutation in our study is quite similar to those in previous studies (Mukasa et al., 2012; Senhaji et al., 2016; Wang et al., 2016). However, the percentage of mutations in the astrocytoma group in our study (16.5%) is much lower compared to the previous study in China (60.6%) (Wang et al., 2016) and in Morocco (63.2%) (Senhaji et al., 2016). 

This study showed that *IDH1* mutations were found more commonly in grades II and III of glioma. In this study, 30.8% of grades II and III gliomas harbor* IDH1 *mutation compared to 13% in the grades I and IV glioma group. This number, however, is smaller compared to that in the previous study. *IDH1 R132* mutation is reported to occur in 55-80% of grades II and III oligodendrogliomas and astrocytomas (Van den Bent et al., 2010; Uno et al., 2011a). A study in Japan showed that *IDH1* mutation was found in 10% of GBM (Grade IV), 28% of anaplastic astrocytoma (grade III), and 59% of diffuse astrocytoma (grade II) (Mukasa et al., 2012). A study in India also showed that the highest percentage of *IDH1* mutation cases was seen in grade II diffuse astrocytoma (70%), compared to grade III anaplastic astrocytoma (33.33%), grade IV glioblastoma multiforme (GBM) (13.04%), grade II oligoastrocytoma (50%), anaplastic oligoastrocytoma (66.67%), and oligodendroglioma grade II (70%) (Devi et al., 2018). Similar data from Europe revealed that 72.7% of diffuse astrocytomas (WHO grade II) harbor *IDH1* mutation, 64.0% in anaplastic astrocytomas WHO grade III, 82.0% in oligodendrogliomas WHO grade II, 69.5% in anaplastic oligodendrogliomas WHO grade III, 81.6% in oligoastrocytomas WHO grade II, and 66.1% in anaplastic oligoastrocytomas WHO grade III (Hartmann et al., 2009). A study in a southeast Asian population, however, showed that GBM WHO grade IV harbors the highest percentage of mutation (64.3%), followed by grade III anaplastic ependymoma (14.3%), grade III anaplastic oligodendroglioma (14.3%), and grade II astrocytoma (7.14%). That study, however, had a small number of samples, especially in grade II (4 samples) and grade III (5 samples) (Yusoff et al., 2016). 

Our study showed that* IDH1* mutation was associated with lower expression of Ki-67. This is in line with a previous study in 118 patients with primary GBM from China (Yan et al., 2012). This association has been proposed to be caused by the low proliferation rate accompanying *IDH1* mutation (Yan et al., 2012). This association may need further investigation.

A previous study has shown that *IDH1* mutation is associated with methylated *MGMT* promoter (Yan et al., 2012). Our study showed a similar result with *IDH1 *mutation detected in 41.7% of methylated *MGMT*, compared to 11.1 % in the unmethylated group (p=0.032). In our study, glioma with *IDH1* mutation showed a higher *MGMT* promoter methylation compared to glioma without this mutation (55.6% vs. 18%, p=0.032). The role of *IDH1* mutation in oncogenesis has been suggested to be caused by the involvement of this mutation in the inactivation of tumor suppressor genes through promoter hypermethylation (Yan et al., 2012).

Our study showed that patients without *IDH1* mutation have a significantly shorter duration between onset and initial surgery compared to the patients with the mutation (mean 4.64±6.8 months vs. 13.3±16.6 months, p=0.025). A similar result was reported previously (Nobusawa et al., 2009), showing that the mean time from first clinical symptom to the diagnosis of GBM is significantly longer in patients with* IDH1* mutation (15.2 months vs. 3.9 months). This association has been proposed to be caused by the low proliferation rate accompanying* IDH1* mutation. Another study, however, did not observe a difference in interval between initial symptom and surgery between GBM with and without* IDH1* mutation (Ohno et al., 2016). The authors suggested that the result indicated that the proliferation rate of *IDH1* mutated glioma is not different from that of *IDH1* wild type GBM (Ohno et al., 2016). Further studies need to be performed to clarify this issue. 

Our study showed that patients with *IDH1* mutation have a better prognosis. Median OS of* IDH1* mutated group was 42.4 months, much longer compared to *IDH1 *wild type group at 10.5 months (p=0.016). This agrees with previous studies, which show a clear association between *IDH1* mutation and favorable clinical outcome (van den Bent et al., 2010; Qi et al., 2011; SongTao et al., 2012; Yan et al., 2012; Wang et al., 2014; Yang et al., 2015).* IDH1 *mutation has been shown to be associated with better OS in grade III anaplastic oligodendroglioma and anaplastic oligoastrocytoma (van den Bent et al., 2010; Wang et al., 2014), grade IV glioblastoma (SongTao et al., 2012; Yan et al., 2012), grade I glioma (Qi et al., 2011), and grade II glioma (Wang et al., 2014). Some researchers have postulated that increased survival in patients with low-grade glioma with *IDH* mutations is an indication of the influence of *IDH *mutations in response to chemotherapy, not different biological behaviors (Turkalp et al., 2014). Some researchers hypothesize that *IDH1/2* mutations consume NADPH and alpha-ketoglutarate, which, in normal conditions, prevent cells from experiencing oxidative stress, thereby increasing sensitivity to radiotherapy (Lee et al., 2007; Fu et al., 2010; Kloosterhof et al., 2011). Zhu (2011) reported that the presence of *IDH* mutations might provide a protective mechanism in glioma patients by interfering with tumor cell metabolism, making them susceptible to cell death. Indeed, previous studies have shown that gliomas with *IDH1* mutation had a better response to treatment with chemotherapeutic agent temozolomide (Houillier et al., 2010; Hartmann et al., 2011; Kim and Liau, 2012; SongTao et al., 2012; Yang et al., 2015), indicating the role of* IDH1* mutation as a predictive marker. However, several studies also showed improved prognosis regardless of chemotherapy, indicating the role of *IDH1* mutation as a prognostic factor (Van den Bent et al., 2010; Yan et al., 2012; Wang et al., 2014). A study in China reported that* IDH* mutation has prognostic significance with more prolonged survival in the *IDH* mutant group. However, they reported that the mutation could not predict a better response to concomitant chemoradiotherapy in anaplastic glioma (Qi et al., 2011). Further studies need to be performed to elaborate more about this point. 

In conclusion, our study in Indonesian patients with glioma showed that 21.7% of patients with glioma harbor *IDH1* mutations, but no *IDH2* mutations.* IDH1 *mutation is significantly associated with smoking, frontal lobe involvement, oligodendroglial histology, grades II and III tumors, lower *Ki67* expression, methylation of *MGMT *promoter, shorter interval between onset of symptoms and initial surgical interventions, and longer OS. These results suggest that identification of *IDH1 *mutations is an important step in the management of patients with glioma in Indonesia, and should be incorporated in daily clinical practice. Further studies need to be done to elaborate more about the role of *IDH* mutations, including its role in determining patient’s response to treatment such as chemotherapy.
